# Early biomechanical outcome in patients with acetabular fractures treated using the pararectus approach: a gait and stair climb analysis study

**DOI:** 10.1007/s00068-021-01655-7

**Published:** 2021-04-09

**Authors:** Andreas Brand, Christian von Rüden, Carina Probst, Lisa Wenzel, Peter Augat, Mario Perl

**Affiliations:** 1 Institute for Biomechanics, BG Unfallklinik Murnau and Paracelsus Medical University Salzburg, Professor-Küntscher Straße 8, 82418 Murnau am Staffelsee, Germany; 2 Institute for Biomechanics, Paracelsus Medical University Salzburg, Salzburg, Austria; 3 Department of Trauma Surgery, BG Unfallklinik Murnau, Murnau, Germany; 4 Department of Trauma and Orthopaedic Surgery, University Hospital Erlangen, Friedrich-Alexander-University Erlangen-Nürnberg (FAU), Erlangen, Germany

**Keywords:** Acetabular fracture, Pararectus approach, Biomechanics, Gait analysis, Functional outcome

## Abstract

**Purpose:**

Patients with surgically treated acetabular fractures using extensive dissection of hip muscles demonstrate an incomplete biomechanical recovery and limited joint mobility during movement. The purpose of this study was to evaluate the early biomechanical outcome in a series of patients with acetabular fractures treated using the less invasive anatomical pararectus approach.

**Methods:**

Eight patients (48 ± 14 years, BMI 25.8 ± 3 kg/m^2^) were investigated 3.8 ± 1.3 months after surgery and compared to matched controls (49 ± 13 years, BMI 26 ± 2.8 kg/m^2^). Trunk and lower extremity kinematics and kinetics during gait and stair climb were calculated. SF-12 and the Merle d’Aubigné score were used for functional evaluation. Statistical analysis was conducted using Mann–Whitney test and Student’s *t* test. Effect sizes were calculated using Cohen’s d.

**Results:**

No group differences for lower extremity kinematics during walking and stair climbing were found. During walking, patients showed significant reductions (*p* < 0.05) of the vertical ground reaction force (8%) and knee and hip extension moments (29 and 27%). Ipsilateral trunk lean was significantly increased by 3.1° during stair descend while reductions of vertical ground reaction force were found for stair ascend (7%) and descend (20%). Hip extension moment was significantly reduced during stair descend by 37%. Patients revealed acceptable SF-12 physical and mental component outcomes and a good rating for the Merle d’Aubigné score (15.9 ± 1.7).

**Conclusion:**

Patients showed some biomechanical restrictions that can be related to residual deficits in weight bearing capacity and strength of the hip muscles. In contrast, an immediate recovery of mobility was achieved by preserving lower extremity and pelvic movement. Therefore, the pararectus approach can serve as a viable strategy in the surgical treatment of acetabular fractures.

**Clinical trial:**

Trial registration number DRKS00011308, 11/14/2016, prospectively registered.

## Introduction

Acetabular fractures demonstrate an overall incidence of about 2–8% of all fractures and may rapidly lead to long lasting physical limitations and functional disability [1]. Regarding internal fracture fixation, various surgical access strategies such as the ilioinguinal approach, the Kocher–Langenbeck approach and the Stoppa approach have proven to be effective. However, sufficient visualisation of the fracture site to achieve anatomical reduction is always a challenge in these approaches which could have a significant impact on the functional outcome [2]. In this context, the less invasive anatomical pararectus approach suggests an alternative access route to surgically stabilise displaced acetabular fractures. Its anatomical tissue-conserving shape protects important functional structures and demonstrates particular advantages in terms of visualisation compared to other approaches. This allows better possibilities for anatomical fracture reduction, achieving a step-free reduction in more than 90% of cases [3]. Radiological evaluation also showed a comparable outcome with other minimally invasive surgical procedures while achieving shorter operation time [4, 5]. In this context, a significantly improved reduction in joint space with similar complication rates was found, when the pararectus approach was compared to the ilioinguinal approach [6]. A crucial aspect in the rehabilitation process following comminute acetabular fractures is the early recovery of mobility. Current surgical interventions that require extensive dissection of major hip muscles demonstrated to have a negative impact on walking quality [7, 8]. Although the pararectus approach indicates to be a promising anatomical access to address acetabular fractures involving the quadrilateral plate, clinical studies on the early postoperative biomechanical outcome and functional mobility are still missing. Instrumented gait analysis can serve as a viable quality tool in the objective assessment of human movement in a clinical setting [9]. In so far, acetabular fractures using the anterior ilioinguinal or the posterior Kocher–Langenbeck approach demonstrated incomplete recovery of hip and pelvic biomechanics including limited mobility and an altered functional outcome after surgery [10–12]. However, most of these gait studies examined patients more than 12 months after surgery, and the immediate postoperative result, therefore, is only partially reflected. The pararectus approach is expected to result in less muscle damage and due to its minimally invasive character it is intended to achieve an early and fast recovery after surgery [13, 14]. Therefore, the purpose of this case series study was to investigate the early biomechanical and functional outcome of patients after internal acetabular fracture fixation treated using the pararectus approach. We hypothesised, that surgical treatment using the pararectus approach results in an early recovery of biomechanical function that is comparable to a healthy control group. In particular, everyday movement tasks such as walking and stair climbing will be evaluated. We further hypothesised that patient reported outcomes on physical health, mental health, and hip function are comparable to the healthy population.

## Materials and methods

### Study design

A total of eight male patients (age 48 ± 14 years, height 183 ± 4 cm, weight 86 ± 12 kg, BMI 25.8 ± 3 kg/m^2^) with isolated unilateral acetabular fractures were included in this prospective case series study. All fractures were coded as anterior column fractures including displaced quadrilateral plate according to the Judet and Letournel classification [15]. Involved side was right in three and left in five patients. For comparison with the normal population a group of eight age, height, weight and gender matched healthy controls (age 49 ± 13 years, height 181 ± 4 cm, weight 84 ± 10 kg, BMI 26 ± 2.8 kg/m^2^) were also included. All patients were treated by open reduction and internal fixation using the pararectus approach. Quality of fracture reduction was evaluated according to the modified Matta criteria using computed tomography. Patients showed an average joint step of 0.9 ± 0.5 mm that was considered as anatomical [4]. No adverse events such as secondary dislocation or prolonged healing were observed. Further inclusion criteria were an age between 18 and 65 years, acetabular fracture involving the anterior column and a body mass index (BMI) lower than 35 since severe obesity reduces intraoperative visibility. Exclusion criteria included polytrauma and other multiple injuries of the lower extremities as well as degenerative concomitant diseases such as osteoarthritis and any neurological disorders that affect walking ability. Written informed consent was obtained from all patients who met the inclusion criteria. After surgical treatment, all patients completed a standard aftercare period consisting of approximately 6 weeks of partial weight bearing (20 kg) with hip flexion limitation of 60° and subsequent rehabilitation care. To exclude any influence of aftercare treatment on the study results, a uniform in-house post-treatment protocol was applied to all patients. Early after surgery (3.8 ± 1.3 months), when full weight bearing was regained, patients were invited to the outpatient department of the hospital for measurements of functional and biomechanical data.

### Questionnaires

Clinical patient-reported outcome measures were evaluated using the modified Merle d’Aubigné score and the Short Form 12 questionnaire (SF-12). The modified Merle d’Aubigné score is a reliable score for assessments of hip function including the components of pain, gait and mobility with a maximum scoring of 18 points indicating unrestricted function [16]. The SF-12 is a subjective questionnaire comprising of quality of life sections such as vitality, physical function, bodily pain and mental health. SF-12 outcomes were subdivided into physical and mental component scales which indicate an completely healthy state (maximum score) at values of 57 and 61, respectively [17].

### Instrumented movement analysis

**Fig. 1:**
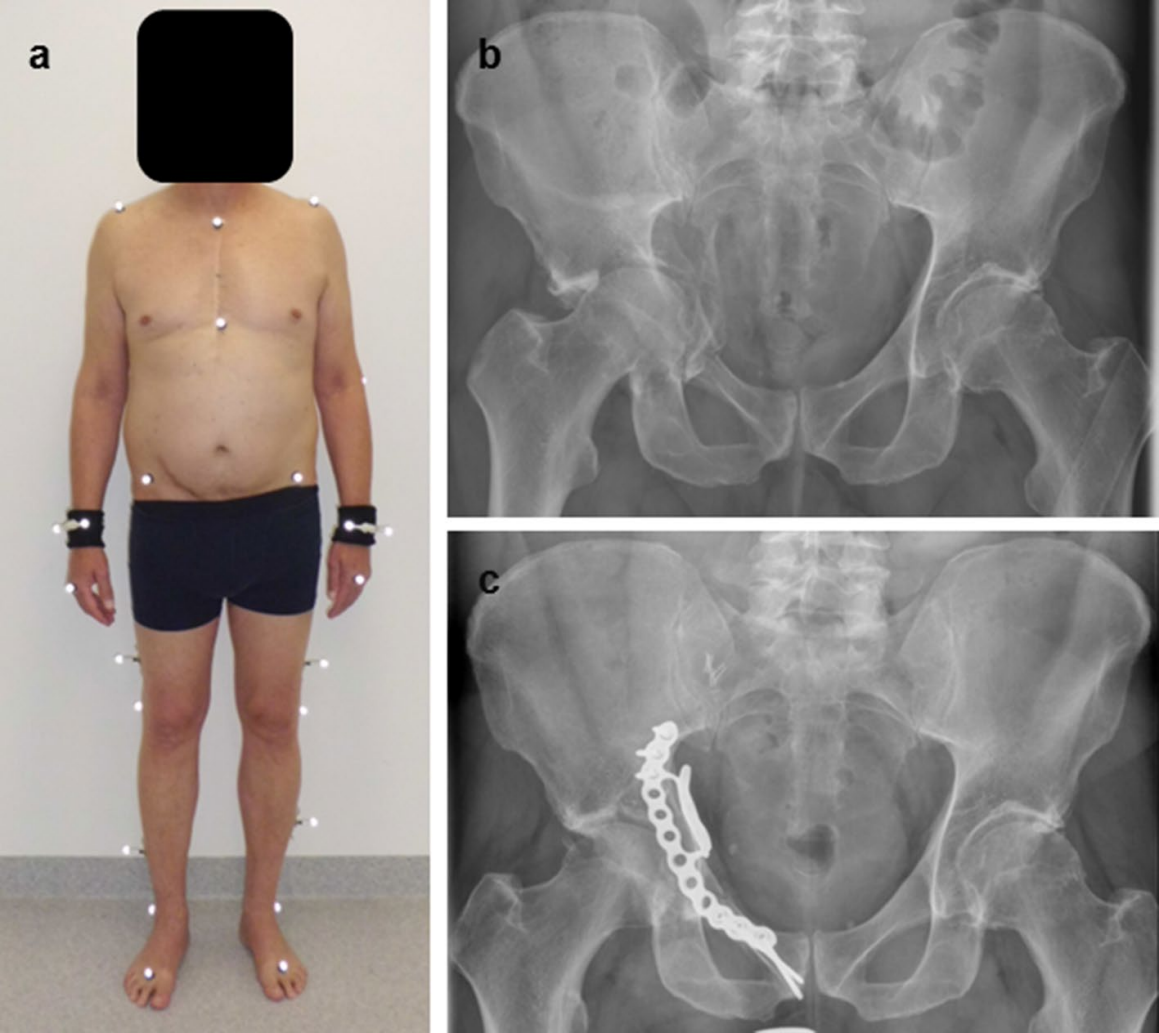
Patient equipped with retroreflective markers for biomechanical follow-up (**a**) following an acetabular fracture involving the quadrilateral plate on the right side treated using the pararectus approach (**b**, **c**). Postoperative radiograph shows the fracture reduced without any step or gap using an anatomically pre-contoured small fragment plate (Stryker PRO system, Stryker Corp., Kalamazoo, MI, USA)

**Fig. 2:**
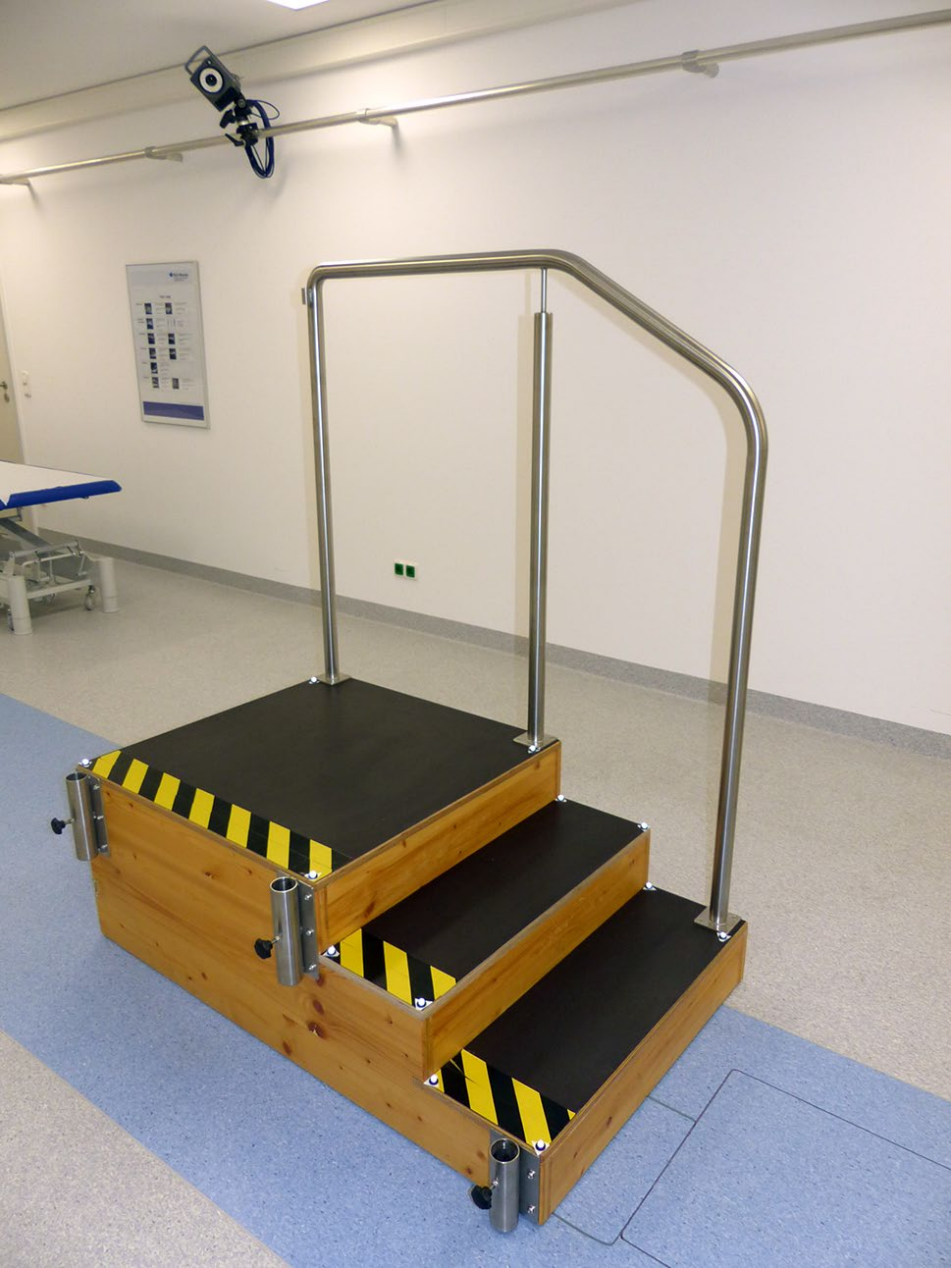
Custom-made stairway scaffold with handrail for biomechanical stair climb analysis

To measure movement biomechanics, an eight-infrared-camera motion capture system (Vicon MX-T20, Oxford, UK) with a sampling frequency of 200 Hz was used. A total of 42 retro-reflective markers were placed on anatomical landmarks according to the protocol of the Conventional Gait Model (Fig. 1) [18, 19]. Kinetic data were obtained using two synchronised embedded force plates (AMTI OR6-7-2000, Watertown, USA). For gait measurements, each individual walked over a 15 m walkway while five valid gait trials (single hit on force plate) were averaged. To measure joint kinematics during stair climbing a custom-made stairway scaffold (German industry standard, DIN 18065) was used. To enable measurements of landing and push-off vertical ground reaction force during stair descend and ascend, the scaffold was positioned in front of the force plates (Fig. 2). As with walking measurements, also a total of five trials were analysed for stair ascend and descend. For kinematic analysis during walking and stair climbing maximum sagittal and frontal movement of the pelvis and the hip as well as sagittal movement of the knee was extracted. To evaluate upper body movement, the maximum ipsilateral trunk lean towards the involved side during stance was measured. Kinetic parameters during walking and stair climbing included maximum values for the sagittal and frontal external hip moment, the sagittal external knee moment and the maximum vertical ground reaction force normalised to body weight. For analysis of gait kinetics, the stance phase was subdivided into a loading phase (initial contact until mid-stance) and push-off phase (terminal stance until pre-swing) as percentage of gait cycle [20]. For data comparison between patients and matched controls, the affected side and one randomised healthy side was used, respectively. Furthermore, spatial–temporal parameters such as walking velocity, cadence, step length, step width and stance duration were compared.

### Statistical analysis

Data comparison of biomechanical parameters and descriptive statistics of functional scores were performed using SPSS version 19.0 (SPSS Inc., Chicago, USA). Biomechanical data were checked for normal distribution using the Shapiro–Wilk test. If normally distributed, the Student’s *t* test for independent samples was used. For non-normally distributed data, the Mann–Whitney test was used to check for significance for those gait datasets and clinical scores. All findings were considered statistically significant at *p* < 0.05. For significant between-group differences, the effect size was calculated using Cohen’s d [21].

## Results

### Questionnaires

The modified Merle d’Aubigné score demonstrated an overall good rating with an average of 15.9 ± 1.7 points. Outcome was excellent (18 points) in one patient, good (15–17 points) in five patients and fair (13–14 points) in two patients. Regarding the SF-12, the mean physical component scale reached 41 ± 8 points while the mental component scale reached 46 ± 10 points.

### Movement biomechanics

During level walking, spatio-temporal parameters of patients with acetabular fractures showed a significant difference with a large effect size for cadence (8%) and walking velocity (14%) when compared to matched controls. No significant differences between both groups were found for stance duration, step width and step length (Table 1).

**Table 1:** Spatio-temporal parameters during level walking in patients after acetabular surgery compared to matched controls. Data is presented as mean (standard deviation)

	Patients	Controls	*p* value	Cohen’s d
Walking velocity (m/s)	1.2 (0.1)	1.4 (0.2)	**0.01**	1.2
Cadence (steps/min)	106 (8)	115 (5)	**0.01**	1.2
Step length (m)	0.8 (0.3)	0.72 (0.1)	0.38	–
Step width (m)	0.19 (0.04)	0.16 (0.03)	0.15	–
Stance duration (% gait cycle)	60.3 (1.3)	60.7 (1.8)	0.42	–

Bold *p* values indicate a significant between-group difference

**Fig. 3:**
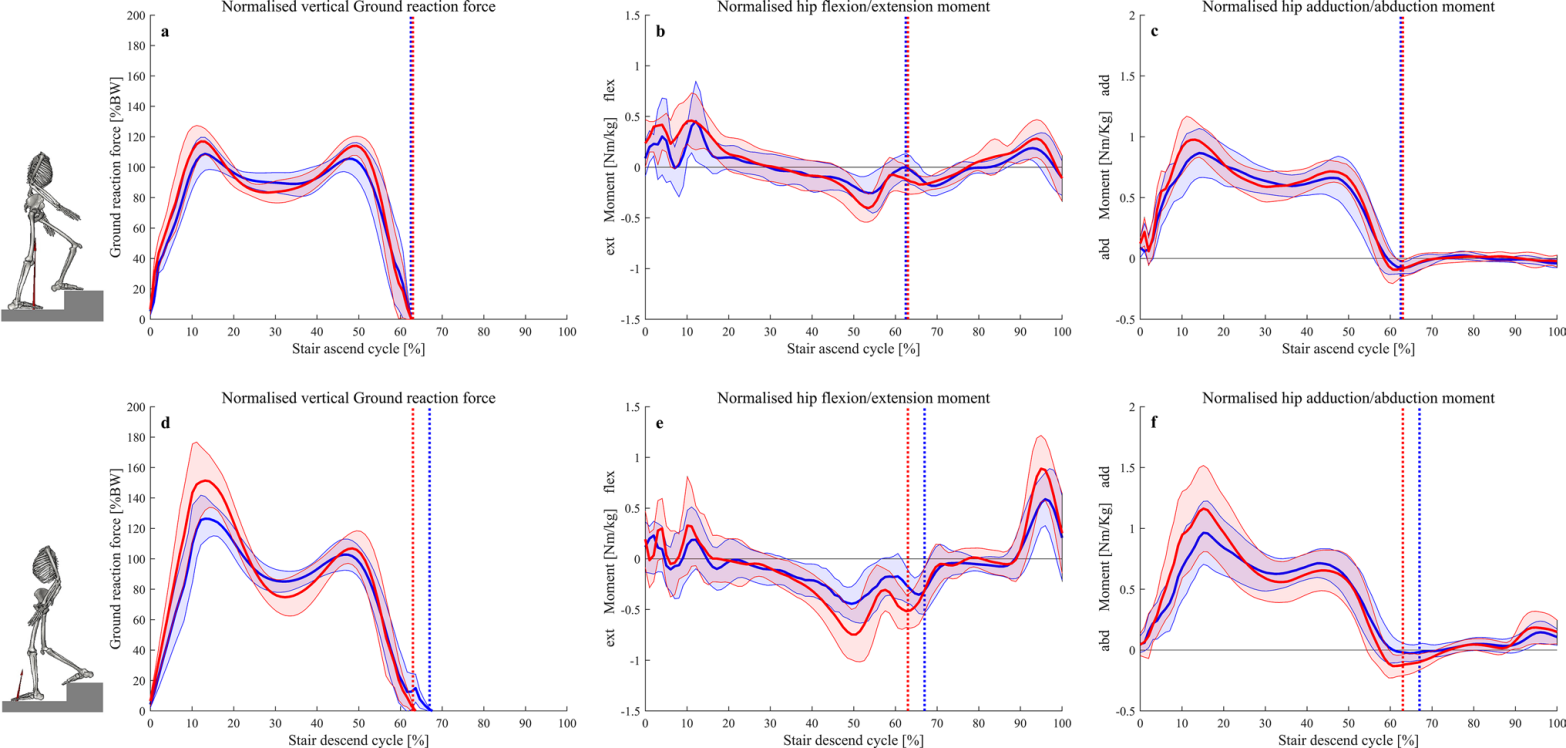
Normalised (BW = body weight) vertical ground reaction forces (**a**, **d**), hip flexion/extension moments (**b**, **e**) and hip adduction/abduction moments (**c**, **f**) during stair ascend and descend for the patients affected side (blue) and matched healthy controls (red). Data are presented as mean with standard deviation. Vertical dotted lines describe the end of stance

No significant differences were found for gait kinematics of the trunk, pelvis, hip and knee (Table 2). During loading phase, no significant difference was found for knee and hip moments, while maximum vertical ground reaction force in patients was significantly reduced by 8%. During push-off phase, patients showed significant reductions with large effect sizes of the maximum knee extension moment (by 29%) and the maximum hip extension moment (by 27%) when compared with healthy controls (Table 3). Comparable graph progressions with no significant differences were found for lower extremity kinematics during stair ascend and descend between groups (see Appendix). In contrast ipsilateral trunk lean in patients (3.2° ± 2°) was significantly increased when compared to healthy controls (0.1° ± 1°) during stair descend (*p* = 0.002; *d* = 1.36). For stair ascend, no significant difference for ipsilateral trunk lean between patients (3.3° ± 1.9°) and matched controls (2.4° ± 1.6°) was found. Differences for normalised vertical ground reaction force and hip moments were found between patients and matched controls during both, stair ascend and descend (Fig. 3). Regarding the maximum normalised vertical ground reaction force, patients demonstrated a significant reduction by 7% during stair ascend. For stair descend, patients also showed reductions for the maximum normalised vertical ground reaction force and for the hip extension moment by 20 and 37%, respectively (Table 4).

**Table 2:** Peak values for walking kinematics during level walking for patients after acetabular surgery compared to matched controls. Data is presented as mean (standard deviation)

	Patients	Controls	*p* value
Ipsilateral trunk lean (°)	3.1 (2.1)	1.4 (1.1)	0.06
Ipsilateral pelvic downward obliquity (°)	3 (2.4)	3.1 (1.6)	0.93
Hip flexion (°)	34.5 (3.4)	32.5 (5)	0.61
Hip extension (°)	8.7 (4.4)	13.6 (7.8)	0.07
Hip adduction (°)	4.9 (2.6)	4.7 (3.3)	0.55
Hip abduction (°)	6.7 (3.1)	8 (5.1)	0.98
Knee flexion (°)	58.4 (4.4)	59.4 (4)	0.55

**Table 3:** Joint moments and normalised vertical ground rection force (GRF) during level walking (separated in loading and push-off phase) for patients compared to controls. Data is presented as mean (standard deviation)

	Patients	Controls	*p* value	Cohen’s d
Loading phase				
Knee flexion moment (Nm/kg)	0.47 (0.2)	0.7 (0.2)	0.07	–
Hip flexion moment (Nm/kg)	0.9 (0.5)	1.32 (0.3)	0.06	–
Hip adduction moment (Nm/kg)	0.93 (0.2)	0.96 (0.1)	0.77	–
Vertical GRF (% body weight)	107 (7)	116 (10)	**0.03**	0.9
Push-off phase				
Knee extension moment (Nm/kg)	0.39 (0.1)	0.55 (0.1)	**0.02**	1.1
Hip extension moment (Nm/kg)	0.86 (0.4)	1.18 (0.2)	**0.04**	1
Hip adduction moment (Nm/kg)	0.77 (0.1)	0.77 (0.1)	0.98	–
Vertical GRF (% body weight)	108 (7)	116 (9)	0.09	–

Bold *p* values indicate a significant between-group difference

**Table 4:** Joint moments and normalised vertical ground reaction force (GRF) during stair ascend and stair descend for patients compared to controls. Data is presented as mean (standard deviation)

	Patients	Controls	*p* value	Cohen’s d
Stair ascend				
Vertical GRF (% body weight)	111 (7)	120 (8)	**0.04**	1
Knee flexion moment	0.4 (0.2)	0.5 (0.2)	0.48	–
Knee extension moment	0.4 (0.2)	0.4 (0.3)	0.97	–
Hip flexion moment	0.4 (0.2)	0.6 (0.3)	0.15	–
Hip extension moment	0.3 (0.1)	0.4 (0.1)	0.08	–
Hip adduction moment	0.9 (0.2)	1 (0.2)	0.18	–
Stair descend				
Vertical GRF (% body weight)	127 (13)	159 (19)	**< 0.01**	1.4
Knee flexion moment	0.4 (0.3)	0.5 (0.3)	0.31	–
Knee extension moment	0.3 (0.1)	0.2 (0.1)	0.29	–
Hip flexion moment	0.3 (0.1)	0.4 (0.3)	0.17	–
Hip extension moment	0.5 (0.1)	0.8 (0.3)	**< 0.01**	1.2
Hip adduction moment	1 (0.2)	1.2 (0.3)	0.19	–

Bold *p* values indicate a significant between-group difference

## Discussion

This study investigated the early biomechanical and clinical outcome in a group of patients with isolated unilateral acetabular fracture addressed through the newly described pararectus approach. Several adaptions could be observed in patients treated using the pararectus approach when compared to healthy controls. Relevant differences in spatio-temporal parameters such as a reduction in walking velocity and cadence were found. This may be explained by the fact that patients try to increase gait stability through a reduced gait speed and a lower number of steps. Comparable results were found in patients with acetabular fractures treated using the Kocher–Langenbeck or the ilioinguinal approach, where reductions in cadence and walking velocity were also reported [10]. Interestingly, our early results showed increased values for walking velocity (30%) and cadence (7%) when compared to the three month outcome in Kubotas study [10]. However, data comparison with this study must be considered with caution, since data have been summarised from both, anterior and posterior surgical approaches [10]. Furthermore, walking velocity and cadence also lay in the same range of a study that investigated patients treated using the anterior ilioinguinal approach after a follow-up between 12 and 44 months [11]. This might suggest that the pararectus approach provides an early recovery of spatio-temporal walking function which is likely comparable to the improved long-term outcome of other surgical approaches.

Gait kinematics showed slight movement deviations by an increased anterior pelvic tilt and a reduced maximum hip extension when compared to matched controls. Although these parameters did not demonstrate significant differences, this might indicate a compensatory posture, which serves to protect the ventral abdominal wall muscles under load and to compensate for restrictive hip extension in the early phase after surgery [22, 23]. Pelvic tilt and hip extension 3 months after surgery presented by Kubota et al. showed different results to those reported in our study. Here, pelvic anterior tilt and hip extension both were smaller when compared to their healthy control group [10]. Although several factors such as the rehabilitation process or fracture severity might have an effect, these differences may also refer to the surgical treatment strategy. The pararectus approach uses an anterior incision, which could lead to a slight compensatory movement restriction due to the residual soft tissue penetration in the early phase. Hence, ventral muscles and tendinous tissues might be protected from excessive stretching by an increase in pelvic anterior tilt during walking. In contrast, using a posterior access such as the Kocher–Langenbeck approach might result in weakness of dorsal muscle groups which explain a decrease in pelvic tilt [10].

Regarding kinetic gait parameters that reflect joint load capacity, a reduction of the vertical ground reaction force was found during loading phase, which indicates that patients still had restrictions regarding initial weight acceptance in the first half of stance phase. Significant reductions of maximum hip and knee extension moments during push-off phase compared to the matched healthy controls were also found. Since effect sizes for these differences were large, high relevance can be attributed to these parameters. The reduced hip extension moment during walking can be related to a compensatory strategy to protect the affected side from a higher load transfer and to overcome a residual strength deficit of the hip flexors during push-off phase. In this context, Kubota et al. also demonstrated a significant reduction of the hip extension moment of 13% when compared to their control group 3 months after surgery. In contrast, the hip adduction moment in our study almost coincided between patients and healthy controls which was not the case when other surgical approaches were investigated [10]. This is a very promising result, since a normal hip adduction moment and physiologic frontal pelvis and hip kinematics are important indicators for sufficient hip adductor strength and pelvic control [24, 25]. Lower extremity joint kinematics which were comparable to those of our matched controls further indicate an unrestricted joint mobility in our patients. The lowered knee extension moment during push-off phase might be more related to unloading of the affected side than to muscle strength deficits since this gait phase is mainly characterised by passive knee movement [20]. Furthermore, this might also be attributed to the effect of a lowered walking speed in our patients, which proved to have major effects especially on reducing the sagittal knee joint moment [26].

Lower extremity joint kinematics during stair climb also showed no differences between patients and matched controls. This is surprising, since this movement is more complex and physiologically more demanding than walking on even ground. This indicates a good early biomechanical outcome in our patients, since an unrestricted movement of the hip and knee joint was achieved. Currently no comparable studies dealing with acetabular fractures and stair climb are available. However, patients after total hip replacement show remarkable restrictions of hip motion during stair climbing which indicate a severe functional limitation [27]. In this context, patients in our study appeared to have no restrictions in joint mobility during stair climb at an average of 3 months after surgery. Maximum normalised vertical ground reaction forces of the affected side however were significantly reduced during both, stair ascend and descend. This suggests that despite of an unrestricted joint mobility a relevant deficit in load capacity is still present in the early phase after surgery. It can be assumed that the reduced weight acceptance is mainly due to protect the affected side from higher loads at the hip joint. Especially during stair descend maximum normalised vertical ground reaction force in healthy individuals could easily reach levels of up to 200% of body weight resulting in hip joint contact forces of up to 260% of body weight [28, 29]. In our study, a significantly increased ipsilateral trunk lean toward the involved stance limb was found. This compensatory movement shifts the lever arm toward the centre of the hip to achieve a reduction of the frontal hip moment [30]. However, external hip adduction moments during stair descent were reduced in patients but showed no significant difference to our controls. A larger focus must be put on the significantly reduced hip extension moment during stair descend, which might indicate that some residual deficits in eccentric strength of the hip flexors are still present. During stair ascend vertical ground reaction force was also reduced compared to the matched control group. This was also observed in patients following total hip arthroplasty where vertical ground reaction forces during stair ascend showed reductions in the early phase after surgery [31]. At least in our case series, this could be more related to compensatory unloading of the hip joint than to functional deficits of hip muscles since joint moments were within the same range of controls. In this context, it could be assumed that concentric muscle function of the knee and hip extensors, that is required during stair ascend, showed no restrictions.

Patients demonstrated a lowered clinical scoring regarding self-reported SF-12 physical and mental components. In the early phase after surgery, physical function is reduced by 17% when compared to the age-matched healthy German population (48 ± 8 points). The mental component also revealed a reduction of 10% compared to the healthy German population (51 ± 9 points) [32]. Comparable studies dealing with the surgical treatment of acetabular fractures reported SF-12 physical and mental scores ranging from 40 to 45 points and 55 to 58 points, respectively [33, 34]. At least for the physical component, our results lay within this range which indicates an acceptable functional result already in the early postoperative phase. This is also supported by our results of the modified Merle d’Aubigné score, where patients achieved overall good results. These are also in line with the good outcome after a 2-year follow-up using the pararectus approach. Furthermore, early results for the Merle d’Aubigné score showed a comparable outcome with those of long-term studies using different strategies such as the Kocher–Langenbeck or ilioinguinal approach [35, 36].

### Study limitations

The overall sample size was relatively small and, therefore, might limit the generalisability of our findings due to limited power for the detection of significant differences between groups. Some comparisons almost reached the level of significance (Tables 2 and 3) and it cannot be excluded that these differences might become significant by including more cases. However, variability of biomechanical data within our patient group was low so that movement patterns during walking and stair climbing could be considered as representative. Different walking speed between patients and controls could also have a potential effect on our results, since this parameter proved to have significant effects on gait kinematics and kinetics [37]. Using a patient adjusted walking speed for our matched controls would have been beneficial for data comparison. Another limitation of this study is that no biomechanical long-term adaptions using the pararectus approach were investigated. However, the main study aim was to evaluate gait restoration during the early postoperative phase (up to 6 months after surgery) where full weight bearing is regained and supervised physical therapy is completed. Since no group comparisons regarding other surgical treatments were performed in this study, we also cannot directly state whether the pararectus approach provides a significant advantage to other treatment strategies. In this context, a systematic comparison might be suggested in the future.

## Conclusion

Patients with isolated unilateral acetabular fractures treated using the pararectus approach showed an almost physiologic dynamic joint mobility of the lower extremities and clinically demonstrated a good hip function. However, reduced joint moments and vertical ground reaction forces during walking and stair descend indicate that deficits in weight bearing capacity and dynamic motor control are still present. In this context, a diminished self-reported physical and mental state compared to healthy controls was also observed. Nonetheless, our findings confirm that lower extremity joint mobility during walking and stair climbing can recover in a very early phase after surgery. Thus, the less invasive pararectus approach appeared to be a viable treatment strategy of acetabular fractures to achieve a promising functional baseline for the ongoing rehabilitation process.

## Figures and Tables

**Fig. 4:**
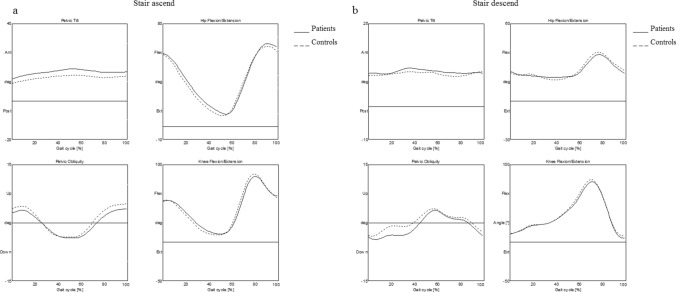
Kinematic graphs of the pelvis and sagittal joint angles of the hip and knee for step over step stair ascend (**a**) and stair descend (**b**)

## Data Availability

The datasets analysed during the current study are available from the corresponding author upon reasonable request.

## Appendix

See Fig. 4 and Tables 5, 6.

**Table 5:** Peak values for kinematics during stair ascend for patients after acetabular surgery compared to matched controls. Data is presented as mean (standard deviation)

	Patients	Controls	*p* value
Pelvic anterior tilt (°)	17.2 (4.9)	14.1 (5)	0.26
Ipsilateral pelvic downward obliquity (°)	4.4 (2.9)	5.5 (2.1)	0.44
Hip flexion (°)	64.2 (4.4)	63.1 (6.2)	0.69
Knee flexion (°)	84.5 (5.4)	89.7 (6.7)	0.14

**Table 6:** Peak values for kinematics during stair descend for patients after acetabular surgery compared to matched controls. Data is presented as mean (standard deviation)

	Patients	Controls	*p* value
Pelvic anterior tilt (°)	12.4 (4.8)	10 (5.4)	0.39
Ipsilateral pelvic downward obliquity (°)	2.1 (2.5)	3.1 (2.4)	0.46
Hip flexion (°)	43.8 (5.6)	43.3 (6.2)	0.87
Knee flexion (°)	87.6 (3.5)	88.8 (5)	0.34
